# Neighborhood Walkability, Walking Difficulties, and Participation in Leisure Activities Among Older People: A Cross-Sectional Study and 4-Year Follow-Up of a Subsample

**DOI:** 10.1177/08982643231191444

**Published:** 2023-07-23

**Authors:** Essi-Mari Tuomola, Kirsi E. Keskinen, Anne Viljanen, Taina Rantanen, Erja Portegijs

**Affiliations:** 1 Faculty of Sport and Health Sciences and Gerontology Research Center, 4168 University of Jyvaskyla, Jyväskylä, Finland; 2 University Medical Center Groningen, Center for Human Movement Sciences, 3647 University of Groningen, Groningen, The Netherlands

**Keywords:** aging, walkability, walking difficulties, leisure activities, geographic information system

## Abstract

**Objectives:** To study cross-sectional and longitudinal associations between objectively assessed neighborhood walkability, walking difficulties, and participation in leisure activities among older people. **Methods:** Self-reported 2 km walking difficulty (intact, modifications, difficulties) at baseline and participating in organized group, outdoor recreation and cultural activities at baseline and follow-up were studied in community-dwelling persons (*N* = 848) aged 75–90. A walkability index, calculated using a geographic information system, was categorized into tertiles (lowest, middle, highest). **Results:** Residence in the highest walkability areas was associated with higher participation in cultural activities and lower participation in outdoor recreation, while the latter was most frequently reported by residents in the lowest walkability areas. Those reporting no difficulties were more likely than those reporting difficulties to participate in all studied activities. Residence in the middle or highest walkability areas predicted higher participation in cultural activities at follow-up. **Discussion:** Older persons activity profiles associate with neighborhood walkability and walking difficulties.

## Introduction

Participating in meaningful leisure activities may provide pleasure, social support, artistic experiences, or a sense of being useful to others, all of which are essential elements of a fulfilling life, including in old age (Rantanen et al., 2021). Earlier research among older people has shown that participation in leisure activities is associated with higher well-being (Adams et al., 2011), better health behavior (Pollack and von dem Knesebeck, 2004), better quality of life (Adams et al., 2011; Silverstein and Parker, 2002), and decreased risk for functional limitations and mortality (Glass et al., 1999; Maier and Klumb, 2005). Leisure activities refer to activities which are pursued for enjoyment or well-being (Verghese et al., 2006) and not related to work or responsibilities of daily living (Verghese et al., 2006).

With increasing age, the match between a person’s walking capacity and neighborhood amenities may become critical for going outside the home and attending activities further away (Skantz et al., 2020a, 2020b). The Selective optimization with compensation (SOC) model proposes that as people age, they must prioritize and optimize their resources to achieve goals while compensating their decreasing abilities (Baltes and Baltes, 1990). The Ecological model of aging by Nahemow & Lawton (1973) posits that an individual’s ability to successfully complete an activity is influenced by the balance between their capabilities and the challenges presented by the environment. According to this model, older adults with fewer resources and declining capabilities are more vulnerable to challenges posed by the environment, which can impact their performance.

Research has shown that older adults with and without walking limitations may experience the same environmental features differently (Sakari et al., 2017; Vaughan et al., 2016; Yang and Sanford, 2012). Walking limitations and declining physical capacity increase individuals’ vulnerability to challenging environmental features and reduce outdoor mobility (Laborde et al., 2022). Outdoor mobility, in turn, is necessary for participation in meaningful leisure pursuits, such as social, cultural, and physical activities (Leyden, 2003; Rantanen, 2013; Sallis, 2009). Outdoor mobility also supports good quality of life and health (Rantanen et al., 2021; Wahl and Weisman, 2003; Wiles et al., 2012). Older adults spend more time in their neighborhood environment than younger age groups (Levasseur et al., 2015). Consequently, the neighborhood environment may enhance or restrict older people’s opportunities to be active outside the home (Sallis, 2009).

However, behavioral adaptations to the demands of their living environment may help older adults to continue engaging in valued activities (Laborde et al., 2022; Rantakokko et al., 2016; Skantz, Rantanen, Palmberg, et al., 2020). With increasing environmental pressure, older individuals may modify their walking to reduce its physiological demands rather than reducing it (Freedman et al., 2017; Nahemow and Lawton, 1973; Skantz, Rantanen, Palmberg, et al., 2020). The first modifications often concern the most challenging physical tasks, such as walking longer distances (Mänty et al., 2007; Weiss et al., 2007). While walking modifications, such as a slower walking pace, resting in the middle of walking, or using a walking aid, may help individuals continue walking to important destinations (Skantz, Rantanen, Palmberg, et al., 2020), they are often also the first signs of functional decline or preclinical disability (Fried et al., 2000). Mobility limitations, including fear of falling, the use of assistive devices (Nilsson et al., 2015) and difficulty walking (Hand and Howrey, 2019), as well as lower daily functional ability (Paillard-Borg et al., 2009; Strain et al., 2002), have been linked to reduced participation in leisure activities outside the home. Specifically, engaging in leisure activities that involve physical activity may be related to an individual’s physical ability to perform such activities (Paillard-Borg et al., 2009; Pritchard et al., 2015).

Neighborhood walkability describes the environment’s suitability for walking to different destinations. Walkability is often operationalized as three features, that is, land use, population density, and street connectivity (Frank et al., 2005; Lovasi et al., 2009). These are often combined to form a walkability index, with a higher value indicating better walkability (Frank et al., 2005). Walkable environments support older people’s independence and mobility, give them an opportunity to maintain social networks, and promote their community engagement (Hassen and Kaufman, 2016). Earlier systematic review has found association between several environmental factors and community participation among older adults (Vaughan et al., 2016). Especially, factors related to walkability, such as high population density (Hand and Howrey, 2019), land use diversity (Beard et al., 2009), and proximity to destinations (Levasseur et al., 2011; Richard et al., 2013) have been associated with community participation and mobility outside the home. In addition, higher walkability has been found to be associated with higher physical activity among older adults (Portegijs et al., 2017; Saelens et al., 2003; Van Holle et al., 2014).

Thus far, only a few studies have focused on neighborhood walkability and participation in leisure activities (Vaughan et al., 2016), and no studies have explored the associations between neighborhood walkability, walking modifications and difficulties, and participation in leisure activities among older adults. While participation in leisure activities may be affected by environmental features, individual factors, such as functional limitations, also likely have a role. The aim of this study was to investigate (1) whether objectively assessed neighborhood walkability at baseline is associated with older adults’ participation in leisure activities outside the home at baseline, (2) whether neighborhood walkability is associated with participation in leisure activities over a four-year follow-up, and (3) how walking difficulties are associated with participation in leisure activities among older people living areas differing in their walkability.

## Methods

This study utilized baseline data gathered for a population-based study entitled “Life-space mobility in old age” (LISPE), which has previously been described in detail (Rantanen et al., 2012). Briefly, a random sample of 2 550 people was drawn from the Digital and Population Data Services Agency and informed about the study. Of these, 848 community-dwelling people aged 75–90 years and fulfilling the inclusion criteria took part. The inclusion criteria were living independently in the municipalities of Jyväskylä or Muurame in Central Finland, being able to communicate, and willingness to participate in the study. At the time of recruitment in 2012, Jyväskylä had about 133 500 inhabitants (the seventh largest city in Finland) and Muurame had about 9 500 inhabitants (Official Statistics of Finland, 2023). The two municipalities have a similar urban structure in which the city and subcenters form the service and residential areas, while the outlying areas vary in residential density. Participant data were collected from in-person at-home interviews in 2012. The LISPE participant data were linked with geographical data from a project entitled “Geographic characteristics, outdoor mobility and physical activity in old age” (GEOage). GEOage located the participants’ home addresses at baseline on a map using the Digiroad dataset (Finnish Transport Agency, 2013) in Geographic Information System (GIS) software ArcMap 10.3 (Esri, Redlands, California, USA). Four years later, a random sample of 298 LISPE participants were invited to take part in the follow-up study MIIA. Of those invited, 77 declined to participate and 15 were not reached. The remaining 206 agreed to take part and thus supplied the four-year longitudinal data. When comparing the MIIA participants (*n* = 206) with the non-participants (*n* = 642) from the original LISPE cohort, there were no differences in terms of sex, number of chronic conditions, or years of education. However, the MIIA participants were found to be somewhat younger and had slightly better cognition and physical performance, as reported by Siltanen et al., (2019). This study combined and analyzed data on the participants and on their leisure activities and walking difficulties, using objectively defined neighborhood walkability.

The Ethical Committee of the University of Jyväskylä approved the study, which was conducted in accordance with the Declaration of Helsinki. Informed consents were obtained from all participants before the assessments.

### Main Variables

#### Participation in Leisure Activities

Participation in leisure activities was self-reported. Activities requiring outdoor mobility were grouped by their social context (organized classes or group activities and clubs vs. individual or small group) (Rantanen et al., 2012) as follows: (1) organized group activities which included participation in class, group or club activities (e.g., choir, physical activity class or church activities); (2) outdoor recreation (e.g., fishing, berry-picking, walking the dog, or gardening); and (3) cultural or other individual activities, including participation in cultural events as a spectator and ad hoc activities (e.g., going to the theater, concerts or a coffee shop). For each question, the frequency response categories were: (1) daily or almost daily, (2) about once a week, (3) two to three times a month, (4) about once a month, (5) a few times a year, (6) rarely, and (7) never. For the cross-sectional and longitudinal analysis, participation frequency was dichotomized as frequently versus rarely based on the distribution and the type of the leisure activity. For outdoor recreation and organized group activities the category “frequently” was defined as participation at least once a week and for cultural or other individual activities at least once a month. The frequency response categories for leisure activities at follow-up were similar to those used at baseline.

#### Perceived Walking Difficulties

In the in-person interview, participants were asked “Do you have difficulty in walking 2 km?” The response categories were (1) able without difficulty, (2) able with some difficulty, (3) able with a great deal of difficulty, (4) unable without the help of another person, and (5) unable to manage even with help. To identify participants using walking modifications, participants who reported being able to walk two kilometers were asked an additional question: “Have you noticed any of the following changes when walking two km due to your health or physical functioning?”. The walking modifications were walking slower, resting during walking, using an aid, having reduced the frequency of walking, and having given up walking distances of two kilometers. For each modification, the participant reported whether they were using that modification (yes/no). For the analyses, participants were categorized into three groups: (a) intact walking (reporting no difficulties or modifications), (b) walking modifications (reporting no difficulty and ≥1 modification), and (c) walking difficulty (reporting at least some difficulty).

#### Neighborhood Walkability

A walkability index, modified from Frank et al. (2004), was created in the GIS. The walkability index, which consisted of land use mix, street connectivity and population density, was calculated within a radius of one kilometer from the participant’s home (Portegijs et al., 2017). The land use mix describes the heterogeneity in the distribution of land use types within the one km circular buffer area (dry land area only) around the participant’s home (Portegijs et al., 2017). Residential areas, services, sport and leisure facilities, and forest and semi-natural areas (built and natural green spaces), were considered in defining the land use mix value (Finnish Environment Institute, 2012). Street connectivity was quantified as the number of intersections along walkable ways within a one-km buffer zone around the home (Finnish Transport Infrastructure Agency, 2013). Only three- or more-way intersections were included and street intersections within 10 m of each other were merged for the calculations. The road network analysis only included walkable ways and thus excluded motorways, trails, winter roads, railroads and ferries over water were excluded from the road network. Population density was defined as the absolute number of residents in the one-km squares of the study areas in which the participants resided (Official Statistics of Finland, 2011). To obtain the walkability index, z-scores were calculated for land use mix, street connectivity, and population density, and summed. Higher index scores indicate better walkability. For the analyses, walkability was categorized into tertiles as lowest, middle, and highest.

### Covariates

Based on previous studies, variables considered likely to correlate with the independent and dependent variables were included as covariates. Participants’ age and sex were obtained from the Digital and Population Data Services Agency as part of participant recruitment. During the home interview, participants were asked to report their total number of years of education. Years of education was used as an indicator of socioeconomic status. The number of self-reported physician-diagnosed chronic diseases was collected using a list of 22 chronic conditions and an open-ended question. Cognitive function was assessed using the Mini-Mental State Examination (MMSE) (Folstein et al., 1975). The MMSE contains 30 items and scores ranges from 0–30. A higher score indicates better function.

### Statistical Analyses

Descriptive characteristics of the participants were compared between those living in the three different neighborhood walkability areas, using Kruskall–Wallis test or Chi-square test, depending on variable distribution. Similarly, participant characteristics were reported as medians and interquartile ranges (IQR) or as percentages. Logistic regression models were used to calculate odds ratios (OR) and 95% confidence intervals for participation in leisure activities at baseline and at the four-year follow-up. Cross-sectional binary logistic regression models were conducted with leisure activity categories as dependent variables and neighborhood walkability and walking difficulties and their interaction as independent variables. Three models were constructed for the cross-sectional and longitudinal analyses for each leisure activity category. In the cross-sectional analyses, the first model tested the association between walkability and participation in a leisure activity (Model 1). To test the role of walking difficulties, it was added to the model (Model 2). Finally, years of education, MMSE score, and number of chronic conditions were added to the model (Model 3). All models were adjusted for age and sex. In addition, the interaction between walkability and walking difficulties was tested and the analyses were adjusted for age, sex, years of education, MMSE score, and number of chronic conditions.

In the longitudinal regression models, participation frequency in leisure activities at follow-up was regressed on neighborhood walkability and perceived walking difficulties at baseline. In the first model, we tested how walkability predicted frequent participation in a leisure activity at follow-up (Model 1). In the second model, we included walking difficulties in the analyses (Model 2) and in the final model (Model 3) we added years of education, MMSE score, and number of chronic conditions. All models were adjusted for age and sex. Finally, sensitivity analyses were conducted to check whether potential changes in the participants’ living environment due to a permanent move affected any associations found. During follow-up, nine participants moved but only for three participants walkability area changed. The results remained similar after excluding these three participants from the analyses (data not shown). SPSS Statistics for Windows (version 26.0; IBM Corp, Armonk, NY, USA) was used for all statistical analyses and statistical significance was set at *p* < .05 in all tests.

## Results

### Participant Characteristics

Characteristics of the full baseline sample and subsample are presented in Table 1. In the full baseline sample, participants living in the lowest walkability area were younger (*p* = .003), had a lower MMSE score (*p* < .001), had a lower level of education (*p* < .001), were more often men (*p* = .001) and more rarely participated in cultural or other individual activities outside the home (*p* = .026) than participants living in the middle or highest walkability areas. Of the 848 baseline participants, 206 participated in the follow-up four years later. At baseline, the subsample participants were younger and had a higher level of education, higher MMSE score, and fewer chronic diseases at the baseline than those who did not participate in the four-year follow-up. No differences were observed between the subsample participants living in the different walkability areas.

**Table 1.** Baseline Descriptive Characteristics by Walkability Tertiles at Baseline for the Full Baseline Sample (*n* = 848) and Subsample (*n* = 206).

	All at Baseline (*n* = 848)				Subsample at Baseline (*n* = 206)			
	Lowest Tertile *n* = 282	Middle Tertile *n* = 284	Highest Tertile *n* = 282	*p*-Value	Lowest Tertile *n* = 74	Middle Tertile *n* = 70	Highest Tertile *n* = 62	*p*-Value
	Median (IQR)	Median (IQR)	Median (IQR)		Median (IQR)	Median (IQR)	Median (IQR)	
Age (years)	79.9 (6.7)	79.7 (7.4)	81.3 (7.7)	**.003** ^a^	79.9 (5.8)	79.3 (8.5)	80.5 (8.0)	.553^a^
Chronic conditions (*n*)	4.0 (3.0)	4.0 (3.0)	4.0 (3.3)	.987^a^	4.0 (3.0)	4.0 (3.3)	4.0 (4.0)	.610^a^
MMSE score	26.0 (4.0)	27.0 (3.8)	27.0 (3.3)	**<.001** ^a^	27.0 (3.0)	27.0 (3.0)	27.0 (2.0)	.551^a^
Education (years)	8.0 (5.0)	9.0 (5.0)	9.0 (7.0)	**<.001** ^a^	8.0 (5.0)	10.0 (5.0)	9.0 (5.0)	.105^a^
	% (*n*)	% (*n*)	% (*n*)		% (*n*)	% (*n*)	% (*n*)	
Men (%)	46.8 (132)	35.6 (101)	31.6 (89)	**.001** ^b^	54.1 (40)	41.4 (29)	33.9 (21)	.055^b^
Walking difficulties (%)	—	—	—	.804^b^	—	—	—	.876^b^
Intact walking	30.5 (86)	31.3 (89)	28.0 (79)		31.1 (23)	37.1 (26)	35.5 (22)	
Walking modifications	27.7 (78)	26.1 (74)	30.5 (86)		31.1 (23)	28.6 (20)	33.9 (21)	
Walking difficulties	41.8 (118)	42.6 (121)	41.5 (117)		37.8 (28)	34.3 (24)	30.6 (19)	
Participation in leisure activities	—	—	—	—	—	—	—	—
Organized group activities (% at least once weekly)	40.1 (113)	43.5 (123)	46.5 (131)	.310^b^	44.6 (33)	47.8 (33)	53.2 (33)	.602^b^
Outdoor recreation (% at least once weekly)	81.1 (228)	78.2 (222)	73.0 (206)	.067^b^	83.8 (62)	82.9 (58)	83.9 (52)	.984^b^
Cultural or other individual activities (% at least once monthly)	27.0 (76)	35.2 (100)	37.0 (104)	**.026** ^b^	28.4 (21)	47.1 (33)	42.6 (26)	.055^b^

*Note.* Statistically significant *p*-values are bolded. Bold values indicate *p* < .05. IQR = interquartile range; MMSE = mini-mental state examination ^a^Kruskall–Wallis test, ^b^Chi-square test.

### Cross-Sectional Associations of Neighborhood Walkability and Walking Difficulties With Participation in Leisure Activities

The logistic regression analyses (Table 2) revealed nonsignificant association between walkability and participation frequency in organized group activities. In Model 1, no statistically significant association was observed between walkability and participation in outdoor recreation. After controlling for the prevalence of walking difficulties, those living in the highest walkability index areas had lower odds for frequent participation in outdoor recreation than those in the lowest walkability areas (OR .61, 95% CI .40–.94). The association remained statistically significant after adjusting for the covariates (OR .60, 95% CI .39–.94). In Model 1, participants living in the highest (OR 1.65, 95% CI 1.15–2.38) or middle (OR 1.47, 95% CI 1.02–2.11) walkability areas were more likely to be frequent attendees at cultural or other individual activities than those living in the lowest walkability area. After controlling for walking difficulties, the associations weakened somewhat but remained statistically significant. Further adjustment for covariates attenuated the odds ratios and the associations became nonsignificant. Those with intact walking and walking modifications attended all the studied leisure activities more often than those with walking difficulties.

**Table 2.** Cross-Sectional Logistic Regression Analyses on Neighborhood Walkability and Frequent (vs. Rare) Participation in Leisure Activities at Baseline (*n* = 848).

	Organized Group Activities						Outdoor Recreation						Cultural or Other Individual Activities					
	Model 1		Model 2		Model 3		Model 1		Model 2		Model 3		Model 1		Model 2		Model 3	
	OR	95% CI	OR	95% CI	OR	95% CI	OR	95% CI	OR	95% CI	OR	95% CI	OR	95% CI	OR	95% CI	OR	95% CI
Walkability tertile																		
Highest	1.26	.90–1.78	1.22	.87–1.74	1.07	.74–1.53	.70	.47–1.06	**.61**	**.40**–**.94**	**.60**	**.39**–**.94**	**1.65**	**1.15**–**2.38**	**1.62**	**1.12**–**2.35**	1.34	.91–1.97
Middle	1.10	.79–1.56	1.10	.78–1.55	1.04	.72–1.47	.87	.57–1.31	.82	.53–1.27	.82	.53–1.27	**1.47**	**1.02**–**2.11**	**1.46**	**1.01**–**2.11**	1.38	.95–2.00
Lowest	1.00	—	1.00	—	1.00	—	1.00	—	1.00	—	1.00	—	1.00	—	1.00	—	1.00	—
Walking difficulties																		
Intact walking			**2.07**	**1.45**–**2.95**	**2.43**	**1.64**–**3.58**			**5.97**	**3.58**–**9.92**	**6.23**	**3.65**–**10.64**			**2.93**	**2.01**–**4.26**	**2.83**	**1.88**–**4.26**
Walking modifications			**1.73**	**1.23**–**2.45**	**1.91**	**1.33**–**2.75**			**3.59**	**2.33**–**5.53**	**3.68**	**2.37**–**5.73**			**1.73**	**1.19**–**2.52**	**1.69**	**1.14**–**2.49**
Walking difficulties			1.00	—	1.00	—	1.00	—	1.00	—	1.00	—	1.00	—	1.00	—	1.00	—
Men (vs. women)	**.69**	**.52**–**.92**	**.63**	**.47**–**.84**	**.63**	**.47**–**.86**	1.18	.84–1.68	.96	.67–1.39	.96	.93–1.01	.82	.60–1.11	**.72**	**.53**–**.99**	**.67**	**.48**–**.93**
Age	.977	.95–1.01	1.00	.96–1.04	1.01	.97–1.05	**.92**	**.89**–**.96**	.97	.93–1.01	.97	.93–1.01	**.95**	**.92**–**.98**	.98	.95–1.02	.99	.96–1.04
Years of education					1.02	.98–1.06					1.01	.97–1.06					**1.06**	**1.02**–**1.11**
MMSE score					**1.09**	**1.03**–**1.15**					.98	.91–1.05					**1.06**	**1.00**–**1.13**
Number of chronic conditions					**1.09**	**1.03**–**1.16**					1.03	.95–1.01					1.02	.95–1.09

*Note.* Values in bold; if the 95% CI does not contain the value 1, *p* < .05. OR = odds ratio, CI = confidence interval, MMSE = mini-mental state examination.

**Figure 1.**
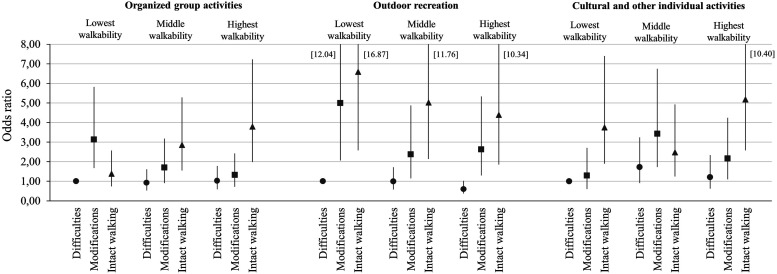
The odds for frequent (vs. rare) participation in leisure activities at baseline by interaction of neighborhood walkability and perceived walking difficulties (*n* = 848).

For the interaction analyses, we formed nine groups based on the combined distribution of walking difficulty and the three neighborhood walkability areas and assigned the participants with walking difficulties living in the lowest walkability tertile as the reference group. Figure 1 presents the fully adjusted odds ratios for frequent participation in leisure activities. Individuals with walking difficulties consistently had the lowest odds for frequent participation in any activity regardless of their neighborhood walkability tertile. For most activities, frequent attendance was most likely among those with intact walking and intermediate attendance among those with walking modifications. There were two exceptions to this: in the lowest walkability areas those with walking modifications had the highest odds for frequent participation in organized group activities, and in the middle walkability areas those with walking modifications had the highest odds for frequently attending cultural or other individual activities. Figure 1 also shows that the odds for frequent participation in outdoor recreation were the highest in the lowest walkability areas. In all, many of the 95% confidence intervals in Figure 1 overlap, indicating a need for interpretive caution.

### Longitudinal Associations of Neighborhood Walkability and Walking Difficulties With Participation in Leisure Activities

No statistically significant associations between neighborhood walkability and frequent participation in organized group activities were observed at follow-up (Table 3). Living in a middle walkability neighborhood increased the odds for frequent participation in outdoor recreation at follow-up in the age- and sex-adjusted model (OR 2.79, CI 1.04–7.50) and in the fully adjusted model (OR 2.92, 95% CI 1.03–8.30). However, after adding walking difficulties into Model 2, the association was attenuated to borderline of significance. Older people living in the middle (OR 3.35, CI 95% 1.51–7.40) or highest (OR 3.42, 95% CI 1.52–7.23) walkability neighborhoods had higher odds for frequent participation in cultural or other individual activities compared those living in the lowest walkability neighborhood. The associations were somewhat attenuated but remained significant in all models. Intact walking at the baseline was associated with frequent participation in cultural or other individual activities (OR 2.85, CI 95% 1.24–6.51) and outdoor recreation (OR 2.92, CI 95% 1.01–8.52) at the four-year follow-up in the age- and sex-adjusted models. After adjusting with covariates, the association between intact walking and participation in cultural or other individual activities remained statistically significant whereas the association between intact walking and participation in outdoor recreation became nonsignificant.

**Table 3.** Longitudinal Logistic Regression Analyses on Neighborhood Walkability and Frequent (vs. Rare) Participation in Leisure Activities at Follow-Up (*n* = 206).

	Organized Group Activities						Outdoor Recreation						Cultural or Other Individual Activities					
	Model 1		Model 2		Model 3		Model 1		Model 2		Model 3		Model 1		Model 2		Model 3	
	OR	95% CI	OR	95% CI	OR	95% CI	OR	95% CI	OR	95% CI	OR	95% CI	OR	95% CI	OR	95% CI	OR	95% CI
Walkability tertile																		
Highest	1.74	.86–3.54	1.61	.79–3.31	1.48	.71–3.08	1.75	.70–4.39	1.49	.58–3.79	1.37	.53–3.55	**3.42**	**1.52**–**7.23**	**3.24**	**1.41–7.43**	**2.96**	**1.25**–**7.02**
Middle	.79	.39–1.62	.73	.36–1.51	.72	.35–1.49	**2.79**	**1.04**–**7.50**	2.59	.95–7.08	**2.92**	**1.03**–**8.30**	**3.35**	**1.51**–**7.40**	**3.12**	**1.39–7.00**	**2.93**	**1.26**–**6.79**
Lowest	1.00	—	1.00	—	1.00	—	1.00	—	1.00	—	1.00	—	1.00	—	1.00	—	1.00	—
Walking difficulties																		
Intact walking			2.12	.99–4.56	1.86	.81–4.27			**2.92**	**1.01–8.52**	2.86	.90–9.13			**2.85**	**1.24**–**6.51**	**2.83**	**1.10–7.28**
Walking modifications			1.47	.69–3.12	1.34	.62–2.93			1.87	.74–4.74	1.86	.70–4.92			1.43	.62–3.32	1.36	.55–3.38
Walking difficulties			1.00	—	1.00	—	1.00	—	1.00	—	1.00	—	1.00	—	1.00	—	1.00	—
Men (vs. women)	.91	.50–1.66	.80	.43–1.49	.77	.41–1.46	1.15	.51–2.60	.97	.42–2.27	.85	.35–2.03	.86	.45–1.64	.70	.35–1.39	.64	.31–1.32
Age	.96	.89–1.03	.98	.90–1.06	.98	.90–1.06	**.84**	**.76–.93**	**.87**	**.79**–**.97**	**.88**	**.79**–**.98**	**.92**	**.85**–**.99**	.94	.87–1.03	**.93**	**.85**–**1.02**
Years of education					1.00	.94–1.08					1.06	.96–1.17					1.01	.93–1.09
MMSE score					1.05	.91–1.21					.94	.79–1.12					**1.32**	**1.10**–**1.60**
Number of chronic conditions					.99	.87–1.13					.98	.83–1.15					1.03	.88–1.19

*Note.* Values in bold; if the 95% CI does not contain the value 1, *p* < .05. OR = odds ratio, CI = confidence interval, MMSE = mini-mental state examination.

## Discussion

Engagement in leisure activities differed between the participants living in the three different walkability living areas. The present findings showed that living in the highest walkability area, such as the city center, was associated with frequent participation in cultural or other individual activities but with lower participation in outdoor recreation. Participants with intact walking or using walking modifications were more likely than those with walking difficulties to participate frequently in leisure activities. In the four-year follow-up, living in a middle or the highest compared to the lowest walkability area predicted higher participation in cultural or other individual activities.

Previous studies have found an association between walkability and community participation, defined as leisure and social activities engaged in outside the home (Vaughan et al., 2016). In this study, older people living in the highest walkability area participated more frequently in cultural or other individual activities such as going to concerts, the theater, or coffee shops. In line with cross-sectional associations, living in the highest walkability area was associated with frequent participation in cultural or other individual activities over the four-year follow-up. Our results may be explained by better access to services and cultural activities in the highest walkability neighborhoods. Neighborhood walkability describes living environments assessed based on residents’ ability to walk to destinations and services (Sallis et al., 2006). Areas such as city centers are typically high walkability neighborhoods as they may offer more services and a wide variety of cultural activities, and hence a greater likelihood of the availability of preferred activities. Access to services may motivate older adults to go out of home and be physically active (Barnett et al., 2017). The present results support those of a previous study which found that neighborhood factors, such as proximity to services and amenities was associated with higher participation of older adults in social activities, such as attending a cultural or sports event or going to a café (Richard et al., 2009).

Our study showed that living in the highest walkability area was associated with lower participation in outdoor recreation. Outdoor recreation typically occurs in natural settings and hence nature and green areas are important for restorative experiences (Andkjær and Arvidsen, 2015; Hinrichs et al., 2019; Keskinen et al., 2018). In our study, outdoor recreation included nature-based activities such as fishing and berry-picking, and other outdoor activities such as gardening and walking the dog. Nature areas may motivate older people to go outdoors and be physically active (Keskinen et al., 2018; Rantakokko et al., 2015). A previous study among older adults showed that lower walkability was associated with higher odds of reporting gardening (King et al., 2017). Outdoor activities, such as gardening, may be relevant in areas of lower walkability with lower residential density and fewer destinations (King et al., 2017). Moreover, these activities may be closer to home.

Previous research has found an association between walkability measures, such as population density, and participation in club activities but not between population density and volunteering or attending meetings of organizations (Hand and Howrey, 2019). However, we found nonsignificant associations between neighborhood walkability and participation in organized group activities, including classes and club activities. It may be that such activities are equally available around municipality, or that participation in organized activities is more dependent on individuals than on environmental features (Hand and Howrey, 2019).

The current findings accord with those of previous studies showing that walking difficulties are associated with lower participation in leisure activities (Hand and Howrey, 2019; Siltanen et al., 2021). In our study, those with intact walking or walking modifications had higher odds of participating frequently in leisure activities than those with walking difficulties. In addition, the older people reporting intact walking at baseline were also more likely to participate frequently in cultural or other individual activities four years later. Mobility limitations directly hinder going out or going further away from home and may eventually increase dependence on needed transportation. Among older people with mobility limitations, transportation is among the most common unmet needs that reduce access to out-of-home activities. (Casado et al., 2011; Shandra, 2021). Individuals are likely to choose activities that are suited to their physical capacity. According to the model of selection, optimization and compensation, older people need to select goals, optimize their resources to achieve those goals, and compensate to maintain functioning (Baltes and Baltes, 1990). Older adults may maintain their way of living by optimizing their mode of action when they start experiencing a decline in their mobility (Saajanaho et al., 2015; Siltanen et al., 2020). In line with this, we found in our earlier study that using modifications, such as assistive devices and slowing down the pace of walking, may help to maintain greater life-space mobility and autonomy in participation outside the home (Skantz, Rantanen, Palmberg, et al., 2020).

Features of the built environment may affect how older adults with mobility limitations experience their surroundings and are able to participate in activities outside the home (Hand and Howrey, 2019). The ecological model of aging highlights that older adults with declining capabilities are more vulnerable to challenges posed by the environment, which can impact their activity (Nahemow and Lawton, 1973). Older adults’ living environment may be an especially important factor for their outdoor participation (Rasinaho et al., 2007). A previous study reported that those with walking difficulties and living in areas of low residential density were less likely to participate in social activities outside the home (Hand and Howrey, 2019), a finding corroborated by our study. Infrastructural mobility barriers, such as poor street conditions, lack of resting places and long distances may restrict older adults’ outdoor mobility (Rantakokko et al., 2015). Living in a high walkability area, such as a city center, may especially support the outdoor mobility of those with walking modifications. Our study suggests that walking modifications may enable more frequent participation in leisure activities irrespective of neighborhood walkability. Environment may provide opportunities to participate in different leisure activities, but also older people may move living areas which offer pleasant activities and support their physical functioning.

The strengths of this study include a large population-based sample of people over age 75 and very little missing information. Moreover, subjective participant data were studied in relation to objective geographical data. A further strength is the longitudinal component with 4-year follow-up data on leisure participation frequency. As the participants were in relatively good health, the results cannot be generalized to community-dwelling adults with poor functioning. Additionally, the rather small study sample in the longitudinal analyses may limit the generalizability of the results and the results should be interpreted with caution. Neighborhood walkability was objectively assessed using data derived from open data sources. A walkability index, although widely used, may not fully reflect individuals’ perspectives on their neighborhood’s environment (Portegijs et al., 2017).

## Conclusions and Future Directions

The present findings suggest that walk-friendly environments may provide opportunities for participation in cultural activities. Participation in cultural activities may be influenced by the availability of and distance to services whereas individual factors, such as mobility limitations, may be more meaningful in activities directly related to physical functioning. Walking modifications may maintain older adults’ involvement in community activities when the environmental features supporting participation are present. It would be important to identify older adults experiencing the first signs of functional decline and find ways to keep them engaged in activity. Future studies could consider how personal environmental preferences and individual resources, such as motivational factors, affect participation and how different environmental factors support participation. In addition, it would be interesting to know more about the locations of leisure activities in relation to the home and its environment. In sum, leisure activities outside the home foster positive experiences and may help with maintaining fitness in old age, thereby underlining the importance of a achieving a good balance between environmental amenities and a individuals’ interests and functional abilities.
